# A rare case of extensive placenta accreta in twin pregnancy after GnRH agonist treatment of adenomyosis

**DOI:** 10.1186/s40738-021-00097-4

**Published:** 2021-03-03

**Authors:** Shilpi Agrawala, Jeevitha Patil, Sukhkamal Campbell, Terri Lynn Woodard

**Affiliations:** 1 Department of Obstetrics and Gynecology, Baylor College of Medicine, 1 Baylor Plaza, Houston, TX 77030 USA; 2 Department of Gynecologic Oncology and Reproductive Medicine, M.D. Anderson Cancer Center, 1515 Holcombe Blvd, Houston, TX 77030 USA

**Keywords:** Adenomyosis, GnRH agonist, Placenta accreta, Live birth, Frozen embryo transfer

## Abstract

**Background:**

Adenomyosis remains an enigma for the reproductive endocrinologist. It is thought to contribute to sub-fertility, and its only curative treatment is hysterectomy. However, studies have documented increased live birth rates in women with adenomyosis who were treated with gonadotropin releasing hormone agonist (GnRHa).

**Case:**

Here we present a case of a 52-year-old woman with adenomyosis who had three failed frozen embryo transfers (FETs) prior to initiating a 6-month trial of GnRHa. GnRHa therapy resulted in a decrease in uterine size from 11.5 × 7.9 × 7.0 cm to 7.8 × 6.2 × 5.9 cm and a decrease in the junctional zone (JZ) thickness from 19 to 9 mm. Subsequently, she underwent her fourth FET, which resulted in live birth of twins. The delivery was complicated by expansive accretas of both placentas requiring cesarean hysterectomy. The final pathology of the placentas demonstrated an extensive lack of decidualized endometrium that was even absent outside the basal plate.

**Conclusions:**

GnRHa therapy in patients with adenomyosis may improve implantation rates after FET. Previous molecular studies indicate that genetic variance in the expression of the gonadotropin releasing hormone receptor (GnRHR) could explain the expansive lack of decidualized endometrium after GnRHa therapy. Further investigations are needed to determine if GnRHa therapy contributes to the pathologic process of placenta accreta.

## Background

Adenomyosis is a pathologic condition characterized by the presence of endometrial glands and stroma within the myometrium. While histopathology is the gold standard for diagnostic confirmation, improved ultrasound and MRI technology has led to highly sensitive and specific alternative modalities for diagnosis. A recent systematic review showed that the sensitivities for ultrasound and MRI, respectively, were 72 and 77% [1]. MRI specificity is slightly higher at 89% compared to 81% for ultrasound. When diagnosing adenomyosis using either imaging modality, physicians focus on the junctional zone, the heterogeneity of the uterine wall, and asymmetry in the thickness of the uterine walls [2–4].

The growing utilization of non-invasive diagnostic imaging in patients undergoing infertility workup has led to an increased recognition of adenomyosis in this population. Due to its association with sub-fertility and the definitive treatment being hysterectomy, adenomyosis has presented a clinical dilemma for reproductive endocrinologists. Thus far, there are no formal guidelines for fertility-sparing treatment of adenomyosis, and current methods are largely based on retrospective case series and case reports [5–9]. Surgical resection, typically reserved for focal lesions, carries an increased risk of uterine rupture [6, 9]. Fertility centers can also treat focal and diffuse adenomyosis with medical therapy, which typically consists of gonadotropin releasing hormone agonist (GnRHa) for 3 to 6 months prior to fresh or frozen embryo transfer [5, 6, 10]. By inducing apoptosis and reducing angiogenesis, GnRHa is thought to decrease the size of the ectopic endometrial glands constituting adenomyosis [5, 11]. Here we present a successful twin live birth in a 52-year-old patient with three prior failed frozen embryo transfers (FETs) who, after GnRHa therapy, underwent FET and achieved clinical pregnancy.

## Case description

The patient presented to our fertility clinic at age 48 as a nulligravida desiring pregnancy. She had a history of fibroids for which she had undergone a myomectomy at an outside institution. The patient reported that the surgeon removed nine fibroids. Of note, the operative report was unavailable for review so the type of myomectomy, size of myomas, and depth of myometrial invasion were unable to be confirmed. After surgery, she was told that she would require pre-labor cesarean delivery if she became pregnant. She reported regular monthly menses and no medical comorbidities. Initial evaluation at our clinic included transvaginal ultrasound (TVUS), antral follicle count (AFC) as well as anti-Mullerian hormone measurement (AMH) and assessment for metabolic syndrome. TVUS revealed three intramural fibroids (the largest 2.5 cm in diameter), none of which appeared to be impacting the endometrial cavity. Her AFC was 3, AMH was 0.52 ng/mL, thyroid function and insulin resistance testing were normal.

At her follow-up visit, a saline-infused sonohysterogram (SIS) was attempted but only partially successful due to cervical stenosis. A left hydrosalpinx was visualized, and diagnostic laparoscopy was pursued. In the operating room, cervical dilation was performed. Subsequently, chromotubation revealed non-patent fallopian tubes, and due to extensive adhesive disease, bilateral Filshie clips were placed in lieu of salpingectomy. No evidence of endometriosis was seen during her laparoscopy, and the adhesive disease was thought to be due to her prior myomectomy. She had a repeat SIS which was successful and a normal endometrial cavity was seen. None of the previously seen fibroids were noted to have a submucosal component. A mock embryo transfer was performed without complication.

In spite of our recommendation to pursue donor eggs, the patient highly desired a trial of in-vitro fertilization (IVF) using autologous oocytes. She underwent ovarian stimulation using 450 IU of Bravelle (urofollitopin) and 150 IU of Menopur (menotropin) daily. GnRH antagonist 0.25 mg was started on stimulation day (SD) 4 to prevent premature follicle maturation. She was triggered on SD8 with 10,000 units of human chorionic gonadotropin when there were 3 follicles > 17 mm in size and estradiol peaked at 922 pg/mL. Four oocytes were retrieved from this cycle, but only one was mature. Intracytoplasmic sperm injection (ICSI) was performed on the mature oocyte; however, there was failed fertilization. After this outcome, the patient desired an emotional break from the IVF process.

Fifteen months later, the patient returned to our clinic to resume fertility treatment. At this point, she agreed to use fresh donor oocytes and undergo FET. The 24 year-old donor underwent an uncomplicated ovarian stimulation and oocyte retrieval. A total of 45 mature oocytes were retrieved. Following ICSI, there were 39 two pronuclear embryos that fertilized normally and were cultured to blastocyst-stage in single step media. Eighteen blastocysts were biopsied and vitrified. Fifteen of the 18 embryos were euploid. The patient underwent another SIS and mock ET, both of which were normal. She started 3 estradiol 0.1 mg patches every other day in preparation for FET. During her ultrasound for a lining check, her endometrial stripe (EMS) was 10 mm, so she started 90 mg of 8% vaginal gel twice daily and 50 mg of intramuscular progesterone daily. Six days after progresterone supplementation, she had her first FET of 1 euploid blastocyst. Her pregnancy test 10 days later was negative.

She decided to pursue another cycle, and after extensive counseling regarding the risks associated with twin pregnancy, the decision was made to proceed with transfer of 2 euploid blastocysts. This transfer resulted in an anembryonic pregnancy, diagnosed by abnormally rising B-HCG and ultrasounds done 2 weeks apart which demonstrated a gestational sac without interval development of a fetal pole. Over the next 2 weeks, her B-HCG was serially monitored, as patient desired expectant management, and her levels were trending downward. The pregnancy failed to evacuate completely after expectant management, and she consented to a suction dilation and curettage. The pathology results were confirmatory of an anembryonic pregnancy.

**Fig. 1:**
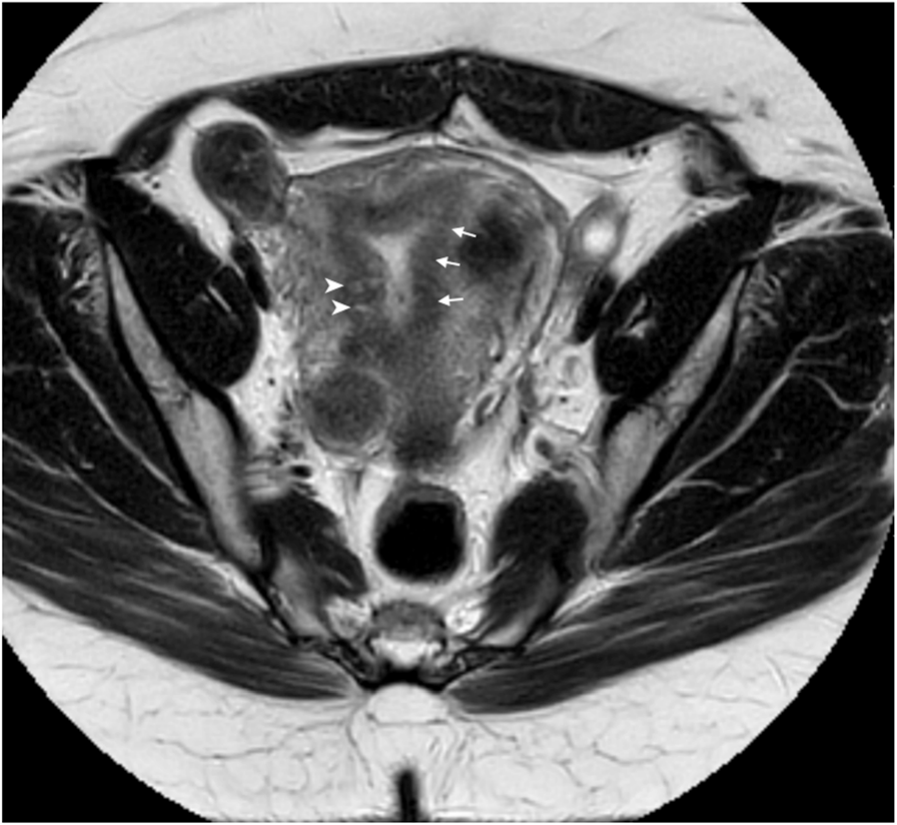
An axial T-1 weighted MRI image shows multiple 1-2 cm myometerial junctional zone cysts (arrowheads) and a thickened junctional zone (arrows)

The patient wished to proceed with another embryo transfer. Prior to this transfer, a pelvic MRI was completed to evaluate if any of her fibroids (described previously) impacted the endometrial cavity. The MRI only showed evidence of adenomyosis as characterized by thickened junction zone (JZ) at 19 mm with multiple 1-2 mm myometrial junctional zone cysts (Fig. 1). Since the endometrial cavity was clear on MRI, the patient underwent a third FET of 2 euploid embryos. Her subsequent pregnancy test was negative.

**Fig. 2:**
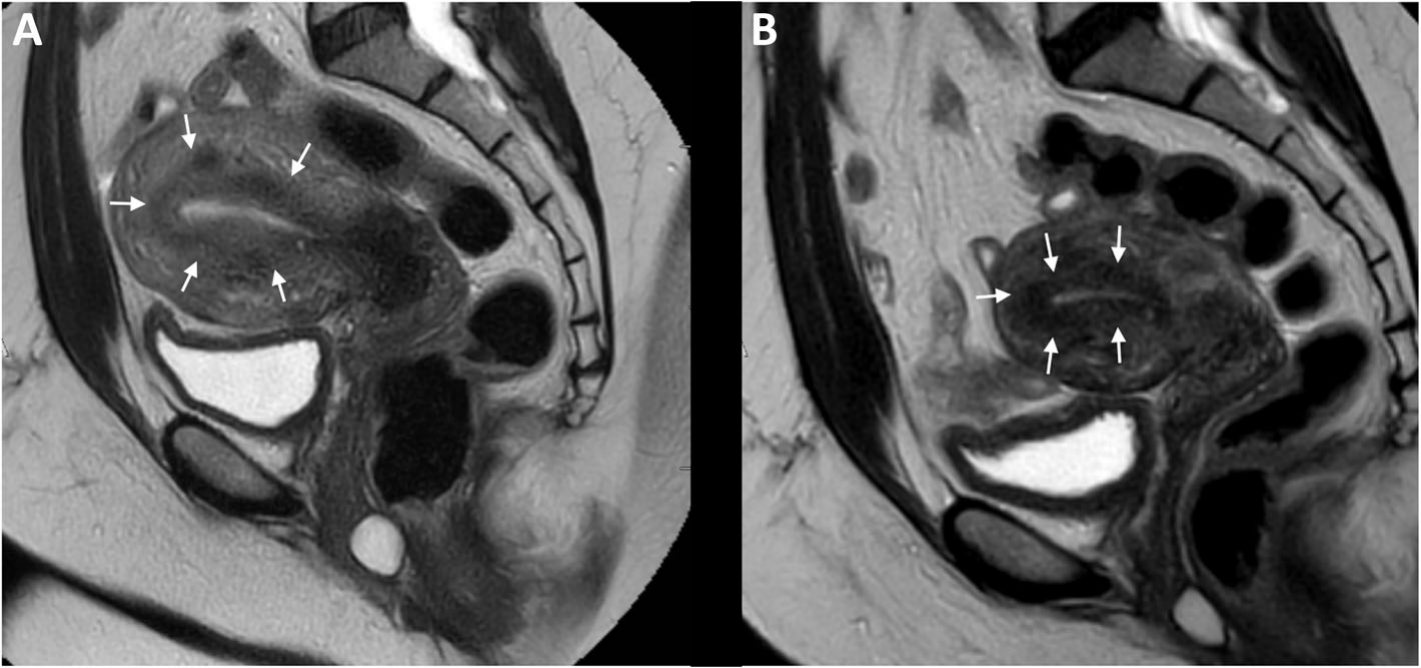
Sagital T-2 images before and after GnRHa therapy showing a decrease in junctional zone thickness. **a** Before GnRHa therapy, the junctional zone is thickened at 19 mm (arrows). **b** After GnRHa therapy for 6 months, the junctional zone has decreased to 9 mm (arrows)

**Fig. 3:**
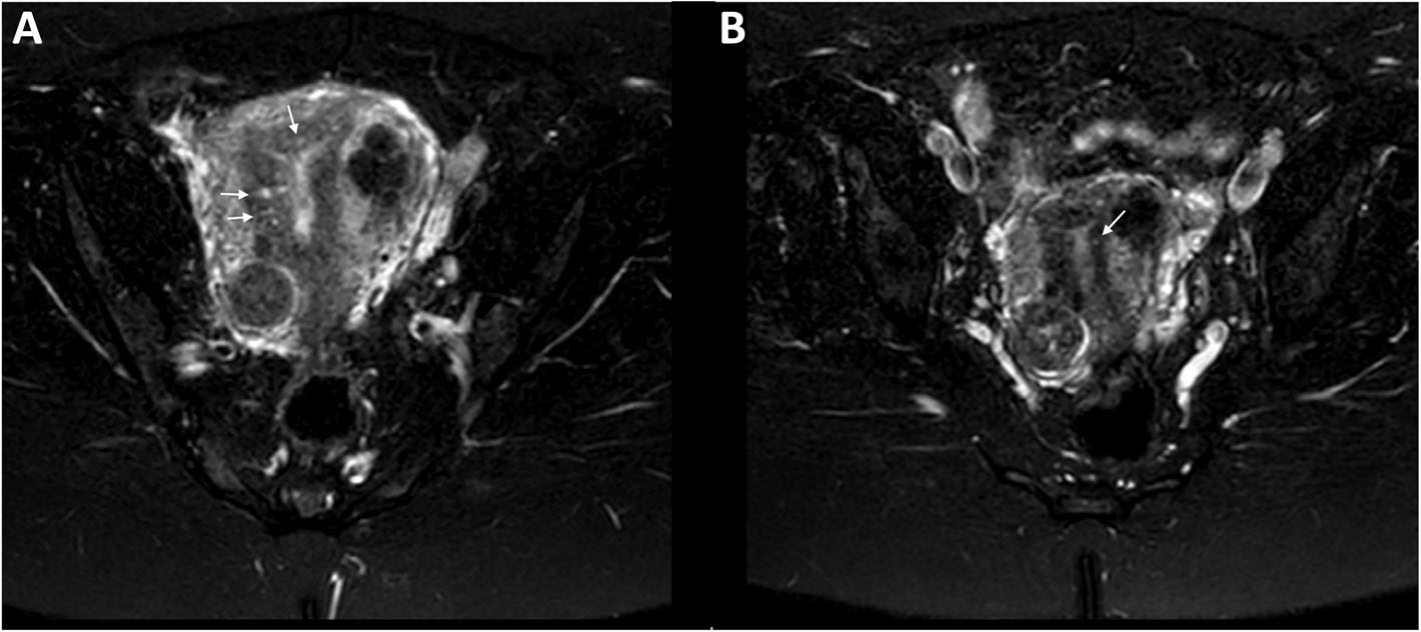
Axial T-2 fat-suppresed images of the uterus before and after GnRHa therapy demonstrating a decrease in cystic spaces in the junctional zone. **a** Cystic spaces (arrows) within the junctional zone consistent with adenomyosis prior to GnRHa therapy. **b** After GnRHa therapy, the cystic spaces (arrow) and junctional zone have decreased in size

After the third unsuccessful FET, the patient was counseled that her adenomyosis could be contributing to the multiple failed FETs. She was counseled on the options of using a gestational carrier vs. medical management. She decided to pursue medical management using leuprolide acetate (GnRHa) for 6 months prior to another FET. She received monthly intramuscular injections of 3.75 mg of leuprolide acetate. Three weeks after the last injection, another MRI was completed which showed a decrease in the JZ from 19 to 9 mm. Uterine size also decreased from 11.5 × 7.9 × 7.0 cm to 7.8 × 6.2 × 5.9 cm (Figs. 2 and 3). Prior to her next FET, the patient was counseled on the number of embryos to transfer. While single embryo transfer (sET) was recommended, we decided to proceed with double embryo transfer due to her history of multiple failed transfers and personal preference. The same protocol for FET preparation was used, and she underwent her fourth FET of 2 euploid blastocysts after an EMS of 10.2 mm was confirmed.

Her initial B-HCG of 1010.3 collected 10 days after FET confirmed pregnancy, and subsequent TVUS several weeks later confirmed viable intrauterine twin pregnancy. She was followed by our fertility center until 10 weeks gestational age (GA) and then transferred to a general obstetrician. Her dichorionic-diamniotic pregnancy was complicated by placenta previa in Twin A for which she had serial ultrasounds. At 27 weeks GA, Twin B was noted to have intrauterine growth restriction (IUGR) < 5th percentile for which she had weekly biophysical profiles. At 31 weeks GA, the patient started developing elevated blood pressures and was transferred to a tertiary care center as the estimated fetal weight of Twin B was less than 1800 g. She was diagnosed with pre-eclampsia without severe features and discharged home after receiving betamethasone for fetal lung maturity. She followed up with the maternal fetal medicine specialists as an outpatient. At 32 weeks, she was admitted for extended maternal fetal monitoring due to worsening pre-eclampsia, IUGR of Twin B < 5th percentile, and placenta previa of Twin A. The patient developed pre-eclampsia with severe features at 33 weeks and was expectantly managed until 34 weeks. She was delivered at 34 weeks via planned primary cesarean due to placenta previa and history of open myomectomy. Cesarean delivery was complicated by extensive accretas of both placentas, which necessitated a supracervical hysterectomy. The patient did not sustain any post-operative complications and has since resumed her daily activities. The neonates also had an uncomplicated hospital stay and are currently meeting all their developmental milestones.

## Discussion and conclusions

This case demonstrates that GnRHa therapy for 6 months in patients with adenomyosis may improve implantation rates after FET, which is consistent with prior studies [7, 8]. Through pituitary downregulation, long-term GnRHa therapy has been shown to decrease the size of adenomyotic lesions [11, 12]. Our patient also had a decrease in her uterine size and a decrease in her JZ from 19 mm to 9 mm on MRI. This decrease in JZ could have facilitated implantation as a thickened JZ prior to FET has been associated with low implantation rates [13]. One study showed that a JZ > 12 mm was associated with an implantation rate of 5% during FET whereas JZ < 10 mm was associated with a 45% implantation rate [14]. Thus, one plausible theory for how GnRHa treatment facilitated pregnancy in our patient was through decreasing the JZ.

The question remains if GnRHa treatment for 6 months also led to the extensive lack of decidualized endometrium, which subsequently led to the development of expansive accretas of both placentas. The patient did have other risk factors for placenta accreta such as IVF treatment, advanced maternal age (AMA), and prior myomectomy [15–19]. The degree of her placental disease raises concern for a a global change to the endometrial stratum basalis, as opposed to the focal changes often seen with IVF pregnancies and a history of myomectomy. The description in the pathology report was that the uterus had no decidualized endometrium on the implantation site of placenta A and the implantation site of placenta B only had a focal area of decidualized endometrium. Within the remainder of the uterine specimen, there was only one other focal area of decidualized endometrium, and it was located within the myometrium, consistent with adenomyosis. There was no histologic evidence of placental invasion past the myometrium nor was there any histologic evidence of endometriosis. Although her prior myomectomy could have contributed to the development of a placenta accreta, it is unlikely to be the sole cause as myomectomies tend to cause focal accretas at the endomyometrial incision site [18, 19]. While undergoing IVF treatments and being of AMA are also risk factors for placenta accreta, neither has been associated with an expansive placenta accreta such as this one. The encompassing surface area of affected endometrium indicates a more systemic defect in decidualization.

Although no studies have investigated whether GnRHa therapy can affect endometrial decidualization, molecular studies have demonstrated that GnRHa also directly exerts anti-proliferative effects on the target organ through the gonadotropin releasing hormone receptor (GnRHR) [11, 20]. In these studies, the direct effect of GnRHa was highly variable between different samples which was attributed to the genetic variance in GnRHR capacity and affinity for GnRHa. We hypothesize that our patient had a genetic variance in GnRHR that could be associated with an increased response to GnRHa. Thus, GnRHa downregulated cellular function at the level of the endometrium so profoundly that it subsequently inhibited or delayed the process of endometrial decidualization. Since this may only affect a small subset of the population, it could explain why other studies of GnRHa therapy in patients with adenomyosis have not shown an increased risk for placenta accreta.

Further investigation is necessary since GnRHa therapy is a widely used medication both in IVF protocols, in treatment of endometriosis, and more recently in patients with adenomyosis. Pregnancies conceived after IVF do have an increased risk for subsequent placenta accreta [15, 16]. These studies did not compare GnRH agonist vs GnRH antagonist protocols, but that could be a potential area of study in the future [15, 16, 21, 22]. Although larger studies have shown that endometriosis is associated with increased risk for placenta accreta, smaller studies did not find an association as it was a rare outcome [23–25]. None of these studies reported whether the patients were treated with GnRHa prior to pregnancy. Case literature has reported that adenomyosis could be a pre-disposing factor for placenta accreta; however, larger studies have not validated that finding [26–29]. Thus, larger studies on patients who receive GnRHa therapy prior to conception for any condition (endometriosis, adenomyosis, or IVF) may be able to demonstrate that there is an increased risk of abnormal placentation with GnRHa therapy.

Lastly, in patients with adenomyosis who receive GnRHa therapy prior to FET, we would recommend single embryo transfer and MFM consultation. We discussed with our patient on multiple occasions that sET is the standard of care when transferring euploid embryos. However, given her multiple rounds of IVF and personal preference, a shared decision was made to transfer two euploid embryos. In addition, we would recommend MFM consultation in patients that achieve pregnancy after GnRHa therapy to monitor for the development of abnormal placentation with serial ultrasounds. Targetted monitoring has been shown to increase rates of detection as well as decrease estimated blood loss and maternal hospital stay [30].

## Data Availability

N/A

## Abbreviations

GnRHa: Gonadotropin releasing hormone agonist
FETs: Frozen embryo transfers
JZ: Junctional zone
TVUS: Transvaginal ultrasound
AFC: Antral follicle count
AMH: Anti-Mullerian hormone
SIS: Saline-infused sonohysterogram
IVF: In-vitro fertilization
SD: Stimulation day
ICSI: Intracytoplasmic sperm injection
EMS: Endometrial stripe
GA: Gestational age
IUGR: Intrauterine growth restriction
GnRHR: Gonadotropin releasing hormone receptor
